# Observance of hygiene and dietary rules and the associated factors among diabetic subjects in Essaouira Province, Morocco: a cross-sectional study

**DOI:** 10.11604/pamj.2022.41.22.30196

**Published:** 2022-01-10

**Authors:** Khaoula Houguig, Nadia Ouzennou, Mahassine Rayadi, Samia Rkha

**Affiliations:** 1 Department of Biology, Pharmacology, Neurobiology, Anthropobiology and Environment Laboratory, Faculty of Sciences Semlalia, Cadi Ayyad University, Marrakech, Morocco,; 2 Higher Institute of Nursing and Technical Health, Marrakech, Morocco,; 3 Endocrinology Service, Sidi Mohamed Ben Abdellah Hospital, Essaouira, Morocco

**Keywords:** Diabetes, hygiene and dietary rules, observance, Morocco

## Abstract

**Introduction:**

hygiene and dietary recommendations are a fundamental pillar of diabetes management. The objective of this study is to measure the rate of observance of hygiene and dietary rules and the factors associated with these among a group of Moroccan diabetic subjects.

**Methods:**

a cross-sectional questionnaire survey was conducted on 522 subjects with types 1 and 2 diabetes followed at different health centres in the province of Essaouira (Morocco).

**Results:**

non-observance of hygiene and dietary rules rate was assessed at 41,4%, (66.5% for diet, 32.4% for lifestyle and 30.8% for physical activity). Rural area (p<0.001), poor glycaemic control (p<0.001), ignorance of hygiene and dietary rules (p<0.001) and long duration of diabetes (p<0.01) are associated with non-observance of diet. Good family support (p<0.01) is associated with good observance of lifestyle recommendations, short duration of diabetes progression (p<0.01) is associated with good observance of physical activity recommendations.

**Conclusion:**

non-observance of hygiene and dietary rules is always a problem in the management of chronic diseases. Ignorance of hygiene and dietary measures, lassitude and difficulty in adapting to a new lifestyle are the main obstacles that diabetics must overcome in order to better manage their disease.

## Introduction

The global prevalence of diabetes in adults has been increasing over recent decades at an alarming pace [1]. In 2017, 451 million adults (ages 18-99 years) lived with diabetes in worldwide. This number was predicted to rise to 693 million by 2045 [2]. There is no country in the world that does not bear some burden from diabetes [1]. According to the International Diabetes Federation (IFD), in 2013, the most popular countries recorded the largest number of deaths: 1271,000 in China, 1065,000 deaths in India, 386,400 in Indonesia, 197,300 in the Russian Federation and 192,700 in the United States of America [3]. Increases in type 2 diabetes incidence generally correlate in general with the globalization, nutrition transition, increasingly sedentary lifestyles, the type of urban growth, etc. [4-6]. Obesity, age, family history, environmental and psychosocial factors, are some risk factors [7-9]. It is the leading cause end-stage kidney disease, blindness and lower limb amputation, and the sixth leading cause of death [10]. The situation in Morocco is as alarming as at the global level. Some national surveys showed the prevalence of type 2 diabetes in Moroccan adults aged 20 years and older varied from 6.6% in 2000 to 12.4% in 2016 [11]. According to the stepwise survey- Ministry of Health 2017-2018. Diabetes is the first cause of blindness, the first cause of end-stage renal disease, the first cause of lower limb amputations [10]. Half of all Moroccan diabetics are disregarding of their disease and the severity of the complications it causes [10]. However, effective management will decrease the incidence of these complications [12]. Indeed, diabetes is a metabolic and nutritional pathology, it occurs as a result of a defect in insulin secretion, insulin action or both [13]. Insulin is what lowers blood glucose levels during the course of the metabolic processes following digestion.

The disease management is based both on pharmacotherapy and on hygiene and dietary rules aimed at controlling blood glucose levels [14]. Achieving this goal reduces the risk of microvascular complications [15]. Morocco, as are all the world's countries, is exerting considerable endeavours to confront this disease. The Moroccan Ministry of Health modulates and focuses its efforts on health education [16]. With this aim in mind, world days have been organised [16], national guides have been developed to help health professionals better manage this disease by providing all necessary information for diabetic subjects to adopt a healthy lifestyle [17,18]. The effective management of diabetes depends on careful observance of advice and recommendations related to drug therapy, diet, physical activity and lifestyle to control blood glucose levels and avoid complications [14]. Diabetics consider these changes as a limitation of their quality of life, which makes them difficult to monitor over the long term [19]. At the global level, several studies have evaluated the factors that may influence, modify, interfere with the follow-up of these recommendations [10-21]. These factors may be related to the socio-demographic profile [22-24], to the treatment [25-27] or to the psychosocial profile [28,29]. In Morocco, and in the face of the inadequacy of work of this kind, particularly in socio-economically vulnerable provinces, there appeared relevant to evaluate the observance of hygiene and dietary rules in diabetic subjects in the province of Essaouira. The objectives of this study are: i)measure the rate of observance of hygiene and dietary rules; ii)identify the factors associated with the observance of hygiene and dietary rules in diabetic subjects in the province of Essaouira.

## Methods

**Study area:** this survey took place in the province of Essaouira, one of the provinces of the Marrakech-Safi region (Morocco). Located to its southwest, it spans an area of 6355 km^2^ with a population of 450,527. It is divided into 52 rural communes and 5 urban communes [30]. Although the province of Essaouira; like the Marrakech-Safi region, has a considerable potential for tourism and agriculture. This region home to the greatest number of poor people in Morocco [31]. This rate hovers around 22.7% in rural areas and 3% in urban areas [32]. However, a larger proportion of economic activity in Essaouira is tied up with tourism and fishing. These two sectors constitute the main sources of income and employment in urban areas, while in rural areas, it is mainly based on goat farming, grain farming and exploitation of Argan trees [33]. The activity rate in the province is estimated at 45.7% [34]. Forty-nine percent of the population is illiterate [35]. Despite concerted efforts to upgrade it (development of rural tourism [36], Argan cooperatives [37] etc.), Essaouira is one of the most vulnerable provinces in the region (vulnerability rate is 22.2%) [38].

**Study design:** a cross-sectional survey was conducted on 522 diabetic subjects followed at 12 health centres in the province of Essaouira between January and December 2018. The health centres provide a basket of health care: monitoring chronic diseases, general medical consultations, nursing, etc. [39]. The management of diabetics consists of medical care and nutritional advice by general practitioners. Based on a review of the literature, the research team developed a detailed structured questionnaire in French. In order to meet the study objectives and to test the hypotheses, cross-cultural adaptation and development of new indicators were necessary. Prior to the validation of the questionnaire, a pre-test was conducted with twenty diabetic participants who resulted in the elimination of questions that seemed redundant or irrelevant. Prior to the validation of the questionnaire, a pre-test was conducted with twenty diabetic participants which resulted in the elimination of questions that seemed redundant or irrelevant. One hundred and five questions were retained instead of the original 124 questions and were organised into six dimensions (20 questions for the Socio-economic characteristics dimension, 21 questions for the diabetes dimension, 27 questions for the nutrition and eating habits, 19 questions for the physical activity and sedentary lifestyle, 14 questions for the well-being dimension and 4 questions for the Anthropometric measurements dimension). The research team chose a single interviewer (a member of the team) who was fluent in French, Arabic and Berber (the respondents' mother tongue) to conduct a face-to-face interview with diabetic subjects who expressed a positive opinion of participating in the study. The interviewer conducts the interview by asking the participants questions, recording the answers and translating the questions into Arabic or Berber if necessary. The interview takes place in a separate room, so that discussion while respecting the privacy of the subject.

The designed questionnaire was used as an instrument to collect detailed data on demographic and socio-economic characteristics (place of residence, age, gender, marital status, education level, occupations, monthly income, etc.), treatment-related characteristics (e.g. age, sex, education, etc.), and the number of patients), treatment-related characteristics (type of treatment, duration of diabetes, comorbidity, complications, etc.), psychosocial characteristics (awareness of health and diet rules, family support, etc.), and compliance with health and diet rules (diet, lifestyle, physical activity). By referring to the Moroccan nutrition guide [17] and to the thematic guide “therapeutic education of type 2 diabetics” [18], this study has assessed the hygiene and dietary rules observance: i)diet: based on the seven classic food groups (beverages, fruits and vegetables, cereals, milk and dairy products, meats, fats, sweet products, medicinal plants have been added because of their undesirable effects), this study has established a list of foods that are not recommended for diabetics and it has noted the corresponding consumption conditions. The interviewee is asked concerning the quantities and frequencies of food consumption in the last four weeks; ii)lifestyle: among the advice offered by the guides, this study chose to collect information related to the sleep of the diabetic, the timing of meals and smoking; iii)physical activity: the interviewee is asked about the nature and frequency of sports activity. The specificities and conditions of observance of each hygiene and dietary rule have been summarised in Table 1. The diabetic subject is considered “observant” if he respects all the conditions of each rule, otherwise, he is “non-observant”. The diabetic subject is considered as “observant” of all hygiene and dietary rules if he respects at least two of the three rules (diet, lifestyle and physical activity).

**Table 1:** conditions for respecting the hygiene and dietary rules

Hygiene and dietary rules		Specificity	Conditions for respecting the hygiene and dietary rules	
**Diet**	Beverages	Tea and coffee	If they are consumed without sugar	The “diet” rule is considered to be observed if all conditions are completed
		Lemonades and alcoholic beverages	If they are not consumed	
	Fruits and vegetables	Fruits and vegetables with a high glycaemic	In case of poor glycaemic control (HbA1c>7%): if they are not consumed	
		index	In case of good glycaemic control (HbA1c<7%):if consumed on an occasional basis	
	Meats	Red meats	If their consumption does not exceed two times a week	
	Fats	Animal fats	If they are not consumed	
		Frying	If they are not consumed	
	Sweet products	Products with a high glycaemic index	In case of poor glycaemic control (HbA1c>7%): if they are not consumed	
			In case of good glycaemic control (HbA1c<7%):if consumed on an occasional basis	
	Medicinal plants		If they are not consumed	
**Life**	Sleep		If the diabetic reports no sleep disturbances	The Rule “healthy life” is considered to be observed if the three conditions are completed
	Timing of meals		If the diabetic reports having meals at set times	
	Smoking		If the diabetic declares that he does not smoke	
**Physical activity**			The rule “physical activity” is considered to be observed if the diabetic practices a sports activity at least 3 times a week for 30 minutes each time	

### Variable definitions

**Professional activity:** the professional activity of the diabetic is defined according to four socio-professional categories (SPC): SPC1: no occupation; SPC2: artisans, employees, workers, shop assistants, farmers, employed workers, labourers, drivers...; SPC3: officials, middle-level professionals...; SPC 4: liberal professions, higher management, major merchants

**Monthly family income:** monthly family income refers to the sum of the monthly wages of all household members at the time of the interview and is expressed in Mad.

**Family support:** the involvement and commitment of the family to support the diabetic to better manage his diabetes were evaluated by asking the following question: are you satisfied with the support you receive from your entourage (spouse, partner, family, friends...)?

**Sample size:**in this study, the minimum required sample size was calculated using the formula:


$$n=Z^{2}{\text{pq/d}}^{\text{2}}$$

where: n = sample size required; Z = Z score for 95% confidence interval = 1.96; p= estimated prevalence of observance of hygiene and dietary rules = 47% (p=0,5, q=1-p); d = margin of error (5% =0.05). The sample was completed to 522 to reduce the risk of non-response.

**Inclusion criteria:** the interviewees were type 1 or 2 diabetics followed in the health centres of the province of Essaouira, whether or not they complied with the hygiene and dietary rules and agreed to participate in the study.

**Exclusion criteria:** diabetic subjects under 18 years of age and pregnant women with diabetes have been excluded from the study.

**Statistical analysis:** data acquisition, calculation of percentages, univariate, bivariate analyses, Khi2 tests were realised by the Statistical package for the Social Science (SPSS) version 20. All statistical tests were performed at the 5% significance level. Observations containing at least one missing data item have been removed. Using the analyses conducted by this software, the factors associated with the observance of each hygiene and dietary rule were identified. On the basis of the observed results, the variables that retained in the logistic regression model have been chosen, in order to determine the weight of the factors associated with the observance of all the hygiene and dietary rules. An adjustment of the variables was necessary for the logistic regression model. This survey considered for the age “less than or equal to 60 years old” against “over 60 years old”, for the instruction level “attend school” against “out of school”, for the monthly family income” <2000 MAD “against” greater than or equal to 2000MAD”, for the type of treatment” taking their treatment “against” without treatment.

**Ethics approval and consent to participate:** the survey was conducted in full respect of local ethical considerations, namely obtaining authorisation N°8874 from the regional and local services of the Moroccan Ministry of Health. The project was approved by the ethics committee (Moroccan Association for Research and Ethics). After informing the diabetic subjects of the purpose of this research, it has sought their consent and agreement to participate in the study. All participants gave their informed consent and all data were collected anonymously.

## Results

**Demographic, socio-economic and cultural characteristics of the interviewees:** five hundred and twenty-two diabetic subjects participated in the survey. They were overwhelmingly female (77.8%), with a sex ratio of 0.28. The age of the surveyed ranged from 20 to 94 years, with an average of 56.6 years (SD = 11.4 years). The age-class distribution showed that the age group between 40 and 60 were the most dominant (56.9%). More than half of those surveyed lived in the urban areas (61.7%). Most subjects had never attended school (82.2% of women and 17.8% of men) [p<0.001], among the literate subjects, only 8% reached secondary education. Moreover, 25.3% of the interviewees were performing a professional activity (41.7% of women and 58.3% of men) [p<0.001]. Eighty-nine percent had a monthly income of less than 2 000 MAD per family (Table 2).

**Table 2:** description of the study population (n=522)

Socio-demographic characteristics			Effective	%
	Age groups (years)	<40	40	7.7
		[40-60]	297	56.9
		>60	185	35.4
	Instruction level	Illiterate	432	82.8
		Primary school	48	9.2
		High school and university	42	8.0
	professional activity	SPC1	390	74.7
		SPC2	118	22.6
		SPC3	12	2.3
		SPC4	2	0.4
	monthly family income	<2 000 MAD	454	87.0
		[2000-4 000 MAD]	47	9.0
		>4 000 MAD	20	3.8
		Married	376	72.0
	Marital	Divorced	28	5.4
	Status	Widowed	100	19.2
		Single	18	3.4
	place of residence	Urban	322	61.7
		Rural	200	38.3
**characteristics of diabetes**	Type of diabetes	NIDD	349	66.9
		IDD	173	33.1
	Treatment	OAD	320	59.8
		Insulin	178	34.1
		without treatment	24	6.1
	Duration of diabetes (years)	<8.4	313	60.0
		>8.4	209	40.0
	Glycaemic control	HbAc1<7%	202	38.7
		HbAc1>7%	320	61.3
	Co-morbidities	No comorbidities	266	51.0
		HBP	131	25.1
		Dyslipidemia	56	10.7
		Others/several	69	13.2
	Complications	No complications	302	57.9
		Retinopathy	54	10.3
		Nephropathy	10	1.9
		Neuropathy	17	3.3
		Others/several	139	26.6
**Psychosocial characteristics**	To be informed about HDR	Yes	68	13
		No	454	87
	Family support	Yes	405	77,6
		No	117	22,4
	Member of a diabetes association	Yes	384	73,6
		No	138	26,4

**SPC:** socio-professional categories: **SPC1**: no occupation; **SPC2:** artisans, employees, workers, shop assistants, farmers, employed workers, labourers, drivers...; **SPC3**: officials, middle-level professionals...; **SPC 4**: liberal professions, higher management, major merchants; **NIDD:** non-insulin-dependent diabetes; **IDD**: insulin-dependent diabetes; **OAD**: oral antidiabetic drugs; **HBP**: high blood pressure, **HDR**: hygiene and dietary rules

**Characteristics of diabetes:** of the total of 522 respondents, 67% had non-insulin-dependent diabetes and more than half were on oral anti-diabetic drugs (59.8%). The mean duration of diabetes was 8.4 years (SD= 6.9 years). Mean glycated haemoglobin was greater than 7% in 61.3% of diabetics. Hypertension and dyslipidaemia were the most common diabetes comorbidities associated (25.1% and 9.6% respectively). Almost 60% of the interviewed had a family history of diabetes. As regards the complications, 10.3% had retinopathy, 3.3% had neuropathy and 1.9% had nephropathy (Table 2).

**psychosocial characteristics:** only 13% of the interviewees were well informed about hygiene and dietary rules. In addition, more than three quarters (78%) of the respondents are supported by their entourage and 74% are members of a diabetes association (Table 2).

### Description of monitoring hygiene and dietary rules

**Figure 1:**
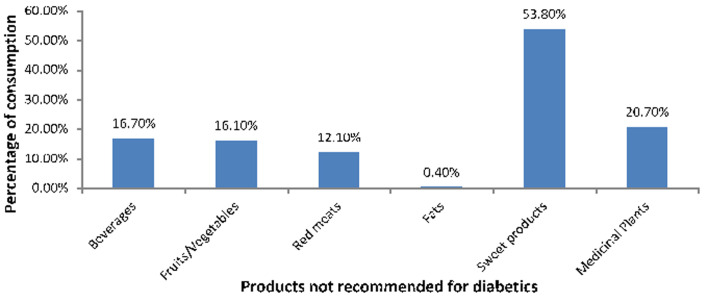
distribution of diabetic subjects who ate foods not recommended for diabetics

**Diet:** the consumption of the different food categories have been evaluated on their adequacy to the reference consumption. Two thirds of the respondents (66.5%) said they had not consumed foods recommended for diabetics: sweetened products (53.8%), sweetened drinks (16.7%) and fruits and vegetables with high glycaemic index (16.1%) were the most foods taken. Figure 1 shows the distribution of diabetic subjects who consumed foods not recommended for diabetics.

**Lifestyle:** the majority of respondents (95.6%) eat their meals at set times and consumed the three main meals of the day, 28% reported having disturbed sleep, only 2.7% who smoked.

**Physical activity:** more than two thirds (69.2%) of diabetic subjects were regularly practising a sports activity, of which 80.1% walked.

**Figure 2:**
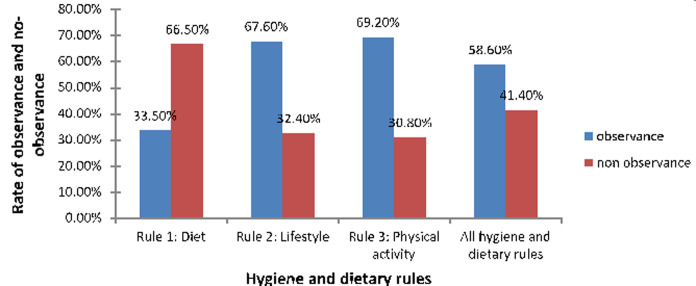
distribution of diabetic subjects according to their adherence to therapy and hygiene and dietary rules

**Observance of hygiene and dietary rules:** Figure 2 shows the overall distribution of diabetic subjects according to their observance or non-observance of hygiene and dietary rules, and shows that observance is good for 58.6% for all hygiene and dietary rules: 33.5% for diet, 67.6% for the lifestyle, and 69.2% for physical activity.

**Factors associated with observance of hygiene and dietary rules:** observance of hygiene and dietary rules has been compared depending on the socio-demographic profile, to the history of diabetes and to the psychosocial factors of the diabetic subject. The variables that were selected were: gender, age, instruction level, monthly family income, place of residence, following treatment or not, duration of diabetes, glycaemic control, to be informed about the disease, family support. The consumption of foods not recommended for diabetics was highly significant in rural rather than urban subjects p<0.001, in subjects with poor glycaemic control (HbAc1>7%) rather than good p<0.001. Respondents who adhered better to diet-related advice were the most informed about their disease p<0.001 and also those who were not on any medication and disease management was based on following hygiene and dietary advice p<0.001. A difference was statistically significant for the duration of the disease p<0.05. Indeed, this survey noted good observance of diet advice in diabetic subjects whose disease duration was less than 8.4 years (Table 3). Observance of lifestyle advice was significant among family-supported subjects p<0.01. A statistically significant difference was also recorded for monthly family income and glycaemic control p<0.05 (Table 3). Regular physical activity was associated with the duration of diabetes p <0.01, place of residence, and gender p <0.05 (Table 3). Binary logistic regression model results reveal that, in order of importance, observance of hygiene and dietary rules is associated with glycaemic control, family support and being informed about the disease (Table 4).

**Table 3:** association of observance with different variables (n= 522)

Variables	Diet			Life			Physical activity		
	Observance No(%) 175(33.5)	Non-observance No(%)	χ^2^/P	Observance No(%) 353(67.6)	Non- observance No(%) 169(32.4)	χ^2^/P	Observance No(%) 361(69.2)	Non-observance No(%) 161(30.8)	χ^2^/P
**Gender**		347(66.5)							
Male	143 (35.2)	263 (64.8)	2.36/NS	274 (67.5)	132(32.5)	0.016/NS	272 (67)	134 (33)	4/*
Female	32 (27.6)	84 (72.4)		79 (68.1)	37 (31.9)		89 (76.7)	27 (23.3)	
**Age groups**									
<40	10 (25)	30 (75)	2.1/NS	33 (82.5)	7 (17.5)	5.3/NS	24 (60)	16 (40)	2.5/NS
[40-60]	106 (35.7)	191 (64.3)		202 (68)	95 (32)		203 (68.4)	94 (31.6)	
>60	59 (31.9)	126 (68.1)		118 (63.8)	67 (36.6)		134 (72.4)	51 (27.6)	
**Instruction level**									
Illiterate	144 (33.3)	288 (66.7)	0.5/NS	289 (66.9)	143(33.1)	0.6/NS	302 (69.9)	130(30.1)	1.15/NS
Primary school	15 (31.2)	33 (68.8)		34 (70.8)	14 (29.2)		33 (68.8)	15 (31.2)	
High school and university	16 (38.1)	26 (61.9)		30 (71.4)	12 (28.6)		26 (61.9)	16 (38.1)	
**Monthly family income**									
<2 000 MAD	145 (31.9)	30 9(68.1)	4.66/NS	308 (67.8)	146 (32.2)	7.8/*	311 (68.5)	143 (31.5)	1.2/NS
[2 000-4 000 MAD]	20 (42.6)	27 (57.4)		26 (55.3)	21 (44.7)		33 (70.2)	14 (29.8)	
>4 000 MAD	10 (50)	10 (50)		18 (90)	2 (10)		16(32.5)8	4 (20)	
**Place of residence**									
Urban	128 (39.8)	194 (60.2)	14.6/**	218 (67.7)	104 (32.3)	0.002/NS	211 (65.5)	111 (34.5)	5.1/*
Rural	47 (23.5)	153 (76.5)		135 (67.5)	65 (32.5)		150 (75)	50 (25)	
**Treatment**									
Taking the treatment	159 (31.9)	339 (68.1)	12.39/***	335 (67.3)	163 (32.7)	0.625/NS	342 (68.7)	156 (31.3)	1.18/NS
Without treatment	16 (66.7)	8(33.3)		18 (75)	6 (25)		19 (79.2)	5 (20.8)	
**Duration of diabetes**									
<8.4 years	118 (37.7)	195 (62.3)	6.1/**	206 (65.8)	107(34.2)	1.17/NS	230 (73.5)	83 (26.5)	6.8/**
≥8.4 years	57 (27.3)	52 (72.7)		147 (70.3)	62(29.7)		131 (62.7)	78 (37.3)	
**Glycaemic control**									
HbAc1<7%	145 (71.8)	57 (28.2)	216/**	147 (72.8)	55 (27.2)	3.9/*	144 (71.3)	58 (28.7)	0.7/NS
**HbAc1≥7%**	30 (9.4)	290 (90.6)		206 (64.4)	114 (35.6)		217 (67.8)	103 (32.2)	
**To be informed about the HDR**									
Yes	38 (55.9)	30 (44.1)	216/***	44 (64.7)	24 (35.5)	0.3/NS	52 (76.5)	16 (23.5)	1.9/NS
No	137 (30)	317 (69.8)		309 (68.1)	145 (31.9)		309 (68.1)	145 (31.9)	
**Family support**									
Good	158 (33.6)	312 (66.4)	0.2/NS	327 (69.6)	143 (30.4)	7.3/**	325 (69.1)	145 (30.9)	0.033/NS
Bad	10 (29.4)	24 (70.6)		16 (47.1)	18 (52.9)		23 (67.6)	11 (32.4)	

**Table 4:** variables of the binary logistic regression model and factors associated with observance of hygiene and dietary rules

	A	E.S	Wald	ddl	Sig	Exp(B)
Gender	0,008	0,258	0,001	1	0,974	1,009
Age	0,210	0,214	0,972	1	0,324	1,234
Instruction level	-0,441	0,288	2,349	1	0,125	0,644
Family monthly income	0,131	0,332	0,156	1	0,693	1,140
**Treatment**	-0,643	0,576	1,244	1	0,265	0,526
Place of residence	0,040	0,214	0,036	1	0,850	1,041
Duration of diabetes	-0,177	0,209	0,716	1	0,397	0,838
Glycaemic control	1,517	0,255	35,473	1	0,000	4,558
To be informed about the disease	-0,660	0,317	4,328	1	0,037	0,517
Family support	0,673	0,234	8,237	1	0,004	1,959
Constante	-3,540	1,242	8,120	1	0,004	0,029

## Discussion

All the recommendations recognise the decisive and fundamental role of hygiene and dietary rules in the management of diabetic conditions. Indeed, good treatment observance, accompanied by a healthy lifestyle can help to manage blood glucose disorders and therefore prevent or delay any complications of diabetes. The UK prospective diabetes study (UKPDS) study showed that a 1% reduction in glycated haemoglobin is associated with a 30% decrease in the relative risk of developing complications, an 18% decrease in the risk of heart attacks and a 25% decrease in the risk of diabetes-related mortality [40,41]. Several difficulties stand in the way of the diabetic to achieve the objectives of treatment in conjunction with the hygiene and dietary rules. This study tried to identify the factors associated with observance of hygiene and dietary rules in diabetic subjects. The lifestyle analysis of the subjects surveyed in this study revealed that the recommendations related to physical activity are the most respected (69%), and subsequently lifestyle (68%), then diet lastly (34%). Similar rates were demonstrated in a national study (52% of respondents had good observance of physical activity while only 35% of diet) [42]. Another national study recorded slightly different rates of observance of diet (47%) and of physical activity (47%) [43]. Observance rates of dietary recommendations are still the lowest. Indeed, the first difficulty encountered by the diabetic is to develop new healthy eating habits. For example, the diabetic to become accustomed to tea without sugar, to minimise bread which was omnipresent in all meals and to dissociate himself from the common dish which constitutes an important marker of the Moroccan cultural identity [44].

It revealed that non-observance of diet is significantly associated with residence in a rural area, ignorance of hygiene and dietary rules, high duration of diabetes and poor glycaemic control. As in all developing countries, in Morocco, belonging to a rural area is associated with a precarious and vulnerable life, a low level of education, difficulty in accessing healthcare, and a lack of specialists and equipment at health care facilities [45,46]. In addition, in the province studied, only one association offers diabetes services to its members, who are predominantly urban (83%). All are obstacles that limit diet observance in rural areas. Diabetic subjects who are informed about their disease and about the hygiene and dietary rules are four times more likely than others to observe the hygiene and dietary rules. Although in urban areas, the majority of the subjects surveyed are members of the diabetes association, but only 13% of them were well informed about hygiene and dietary rules. This rate of ignorance is very worrying and shows all the interest to encourage the creation of other diabetes associations and why not in rural areas, to improve the services and the benefits presented by the association in particular in terms of sensitisation to the hygiene and dietary rules. General practitioners in rural health centres must wear the hats of endocrinologist and nutritionist in parallel to compensate for the insufficiency of these two specialities. In the same context, a study carried out in Bangladesh presented the same results, diabetic subjects who attended therapeutic and nutritional education courses showed good observance of the diet [47]. Another barrier that interferes with adherence to diet recommendations is the problem of lassitude. In fact, in the present study, diabetic subjects whose disease duration is greater than 8.4 years found it difficult to follow diet and physical activity recommendations in the long term. Over time, hygiene and dietary recommendations become routine and the diabetic may no longer pay attention to them. A French study confirmed that lassitude is one of the main obstacles to long-term observance of dietary measures [48]. Calypse and his collaborators presented the same observation “Living with diabetes for more than six years was associated with no change in diet” [49].

Other reasons interfere with regular and adapted physical activity, laziness and pre-existing comorbidities such as cardiovascular disease, osteoarthritis, foot pain and obesity. The reasons for non-observance of physical activity are consistent with those reported in the Bangladeshi study [47]. Other studies have reported other reasons such as fear of hypoglycaemia and lack of time [50,51]. It has been noticed that the respondents whose disease management is based on the respect of hygiene and dietary rules and no medical monitoring was recommended better observe the diet recommendations than subjects whose medication is mandatory. This result is not surprising and leads us to underline the importance of the beliefs and representations of diabetics to their disease. For some, medical treatment, whether oral antidiabetics or insulin, is sufficient and exempts them from following the hygiene and dietary recommendations. In addition, almost 72% of diabetic subjects who strictly observe of diet-related measures recorded good glycaemic control. Moreover, observance of hygiene and dietary measures can only have repercussions on glycaemic control. Several studies have confirmed the improvement of blood glucose levels following a rigorous and relentless monitoring of therapeutic and hygiene and dietary advice [50-55]. In the present study, it was found that diabetic subjects who reported being supported by their entourage, whether it's spouse, parents, children or health personnel, were eight times more likely to comply with hygiene and dietary rules. This shows the importance of improving and promoting the role of the family in the management of the disease [56] by increasing the awareness and information of diabetic subjects and their caregivers about hygiene and dietary rules.

## Conclusion

The identification of factors associated with the observance of hygiene and dietary rules would allow health professionals to adapt and simplify their discourse by inciting diabetics to regularly control their blood glucose which oscillates according to their observance of hygiene and dietary rules, thus directly influencing their physical and psychological health.

**Limitations of study:** in addition to sociodemographic, disease-treatment and psychosocial factors, it would be relevant to add care-related factors such as accessibility to health structures and the health practitioner-diabetic subject relationship.

### What is known about this topic

- Hygiene and dietary recommendations are one of the basic foundations of diabetes management;
- Several factors influence observance of these measures: the sociodemographic and psychosocial profile of the diabetic, and factors related to the disease.

### What this study adds

- No such study has been conducted for the rural population in the province of Essaouira (Morocco);
- This study confirms the positive influence of the respect of the hygiene and dietary rules on the glycaemic control;
- Surveyed subjects not adhering to the diet recommendations were 66.5% and the associated factors were: poor glycaemic control, residence in rural areas, taking the treatment, less informed about the hygiene and dietary rules, disease duration more than 8.4 years; surveyed subjects not adhering to the lifestyle recommendations were 32.4% and the associated factors were: bad family support and poor glycaemic control.
